# Supplementary material for: The intracellular C-terminus confers compartment-specific targeting of voltage-gated Ca2+ channels
Source: bioRxiv. 2023 Dec 23:2023.12.23.573183. Preprint. [Version 1] doi: 10.1101/2023.12.23.573183 (PMC10769351; doi:10.1101/2023.12.23.573183)
Supplement: Supplement 1 [file NIHPP2023.12.23.573183v1-supplement-1.pdf]

bioRxiv preprint doi: https://doi.org/10.1101/2023.12.23.573183; this version posted December 23, 2023. The copyright holder for this preprint (which was not certified by peer review) is the author/funder, who has granted bioRxiv a license to display the preprint in perpetuity. It is made available under aCC-BY 4.0 International license.

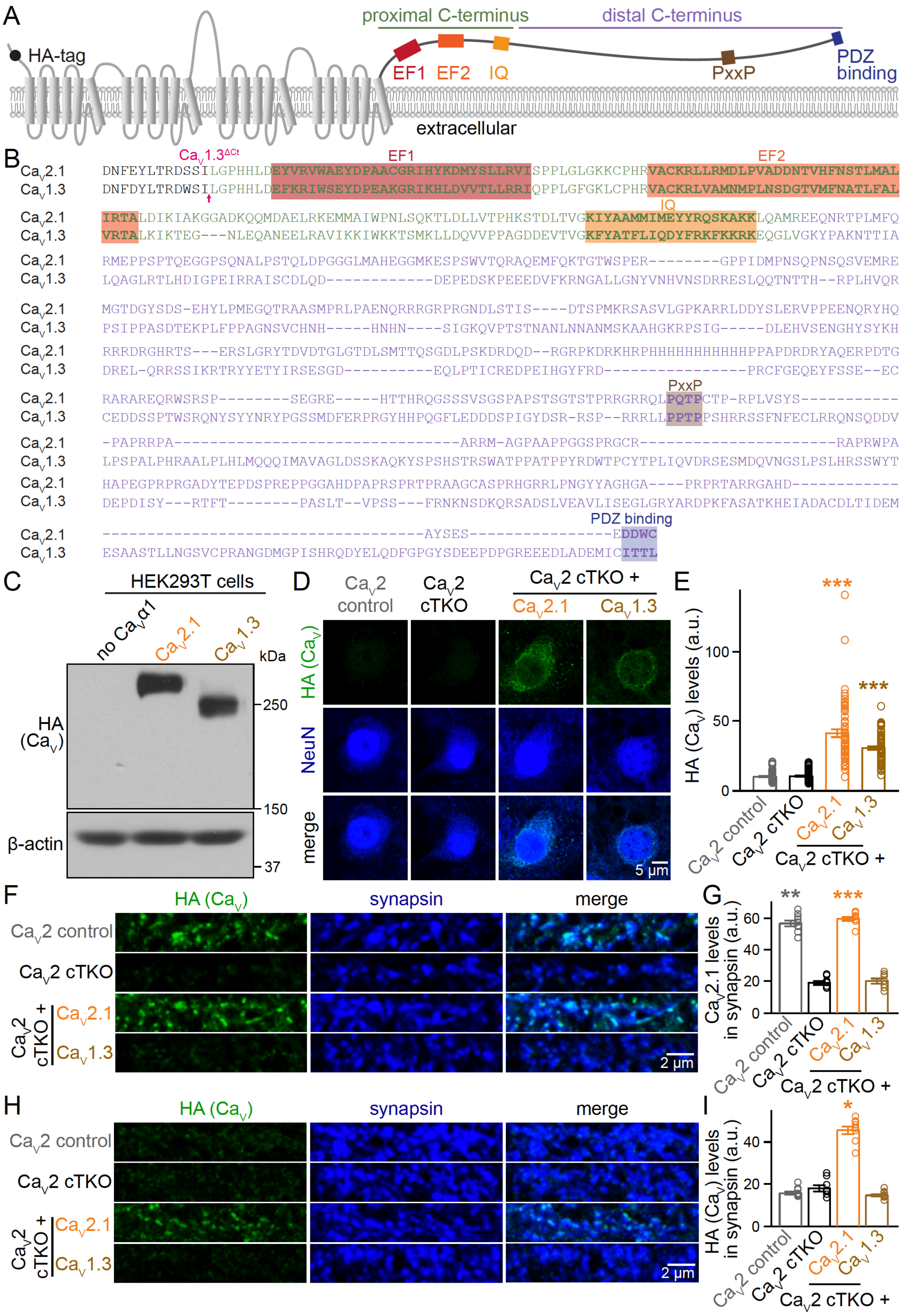

Figure S1. Additional assessment of $Ca_V2.1$ and $Ca_V1.3$ expression and localization.

bioRxiv preprint doi: https://doi.org/10.1101/2023.12.23.573183; this version posted December 23, 2023. The copyright holder for this preprint (which was not certified by peer review) is the author/funder, who has granted bioRxiv a license to display the preprint in perpetuity. It is made available under aCC-BY 4.0 International license.

(A) Schematic of $Ca_V2.1$ with important sequence motifs highlighted, adapted from [20]; EF1 and EF2: EF hands; IQ: IQ motif; PxxP: proline rich motif.

(B) Alignment of the C-terminal sequences starting immediately after the last transmembrane segment (for $Ca_V2.1$, residues DNFE…DDWC are matching with GenBank Entry AY714490.1; for $Ca_V1.3$, residues DNFD…ITTL are matching with GenBank Entry AF370010.1). Sequence motifs are highlighted, and the Ca_V proximal and distal C-terminal segments are labeled in green and purple, respectively.

(C) Western blot of HEK293T cell homogenates after transfection with $Ca_V\beta1$, $Ca_V\alpha2\delta1$, and without (no $Ca_V\alpha1$) or with a $Ca_V\alpha1$ subunit to assess $Ca_V\alpha1$ expression; $Ca_V2.1$ and $Ca_V1.3$ were transfected and analyzed multiple times, but only once in this order.

(D+E) Representative confocal images (D) and quantification (E) of HA levels in cell bodies of neurons stained with antibodies against HA and NeuN. Cell bodies were defined as donut shaped ROIs using the outer edge of the NeuN profile along the main somatic compartment not including the neurites, and by excluding the EGFP-labeled nucleus; 60 somata/3 independent cultures each.

(F+G) Representative areas of confocal images (F) and quantification (G) of $Ca_V2.1$ levels in synapsin ROIs (the imaged areas are identical to the STED scans in Fig. 1C-E); Ca_V2 control, 9 images/3 independent cultures; Ca_V2 cTKO, 8/3; Ca_V2 cTKO + $Ca_V2.1$, 9/3; Ca_V2 cTKO + $Ca_V1.3$, 8/3.

(H+I) As in F and G, but for neurons stained with antibodies against HA, PSD-95 and synapsin (the imaged areas are identical to the STED scans in Fig. 1F-H); Ca_V2 control, 9/3; Ca_V2 cTKO, 8/3; Ca_V2 cTKO + $Ca_V2.1$, 9/3; Ca_V2 cTKO + $Ca_V1.3$, 9/3.

Data are mean ± SEM; $*p < 0.05$, $**p < 0.01$, and $***p < 0.001$. Statistical significance compared to Ca_V2 cTKO was determined with Kruskal-Wallis tests followed by Dunn's multiple comparisons post-hoc tests for the proteins of interest in E, G, and I.

bioRxiv preprint doi: https://doi.org/10.1101/2023.12.23.573183; this version posted December 23, 2023. The copyright holder for this preprint (which was not certified by peer review) is the author/funder, who has granted bioRxiv a license to display the preprint in perpetuity. It is made available under aCC-BY 4.0 International license.

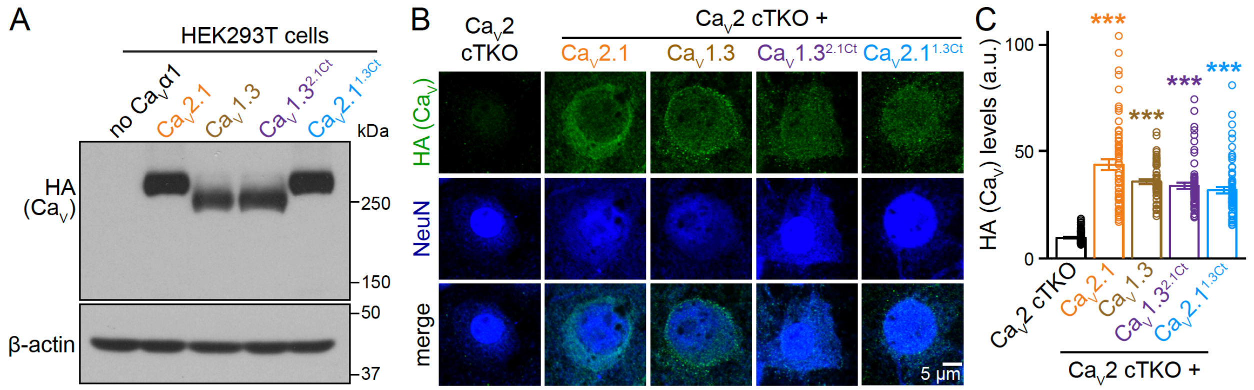

Figure S2. Additional assessment of $Ca_V1.3^{2.1Ct}$ and $Ca_V2.1^{1.3Ct}$ expression.

(A) Western blot of HEK293T cell homogenates after transfection with $Ca_V\beta1$, $Ca_V\alpha2\delta1$, and without (no $Ca_V\alpha1$) or with a $Ca_V\alpha1$ subunit to assess $Ca_V\alpha1$ expression, a representative blot from three independent repeats is shown.

(B+C) Representative confocal images (B) and quantification (C) of HA levels in cell bodies of neurons stained with antibodies against HA and NeuN; 60 somata/3 independent cultures each. Data are mean ± SEM; ***$p < 0.001$. Statistical significance compared to Ca_V2 cTKO was determined with Kruskal-Wallis tests followed by Dunn's multiple comparisons post-hoc tests for the protein of interest in C.

bioRxiv preprint doi: https://doi.org/10.1101/2023.12.23.573183; this version posted December 23, 2023. The copyright holder for this preprint (which was not certified by peer review) is the author/funder, who has granted bioRxiv a license to display the preprint in perpetuity. It is made available under aCC-BY 4.0 International license.

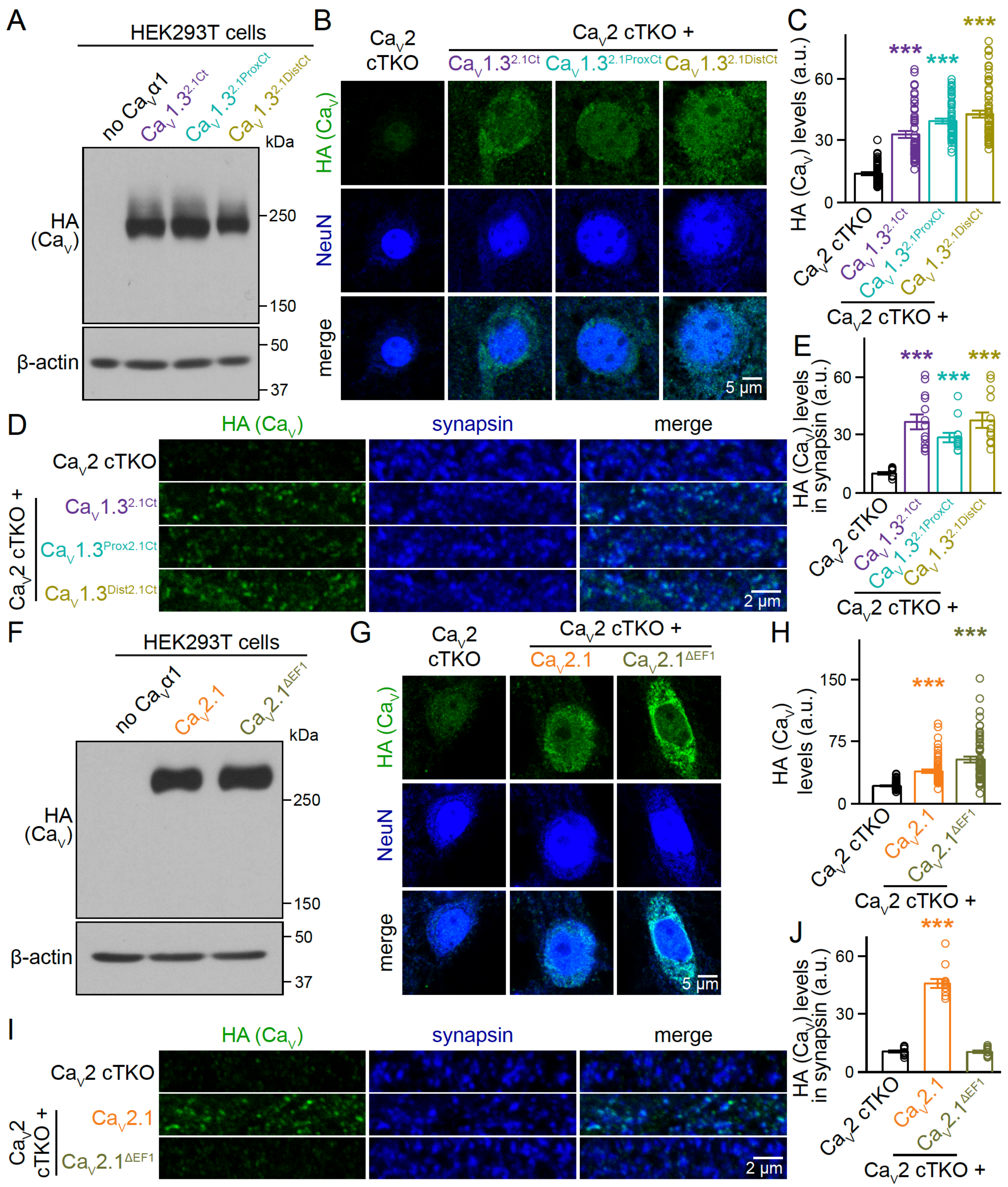

Figure S3. Additional assessment of Ca$_V$1.3$^{2.1ProxCt}$, Ca$_V$1.3$^{2.1DistCt}$ and Ca$_V$2.1$^{\Delta EF1}$ expression and localization.

(A) Western blot of HEK293T cell homogenates after transfection with Ca$_V$β1, Ca$_V$α2δ1, and

bioRxiv preprint doi: https://doi.org/10.1101/2023.12.23.573183; this version posted December 23, 2023. The copyright holder for this preprint (which was not certified by peer review) is the author/funder, who has granted bioRxiv a license to display the preprint in perpetuity. It is made available under aCC-BY 4.0 International license.

without (no $Ca_V\alpha1$) or with a $Ca_V\alpha1$ subunit to assess $Ca_V\alpha1$ expression, a representative blot from three independent repeats is shown.

(B+C) Representative confocal images (B) and quantification (C) of HA levels in cell bodies of neurons stained with antibodies against HA and NeuN; 60 somata/3 independent cultures each.

(D and E) Representative areas of confocal images (D) and quantification (E) of HA levels in synapsin ROIs (the imaged areas are identical to the STED scans in Fig. 3B-D); Ca_V2 control, 9 images/3 independent cultures; Ca_V2 cTKO, 12/3; Ca_V2 cTKO + $Ca_V1.3^{2.1Ct}$, 13/3; Ca_V2 cTKO + $Ca_V1.3^{2.1ProxCt}$, 12/3; Ca_V2 cTKO + $Ca_V1.3^{2.1DistCt}$, 12/3.

(F) Western blot of HEK293T cell homogenates after transfection with $Ca_V\beta1$, $Ca_V\alpha2\delta1$, and without (no $Ca_V\alpha1$) or with a $Ca_V\alpha1$ subunit to assess expression, a representative blot from two independent repeats is shown.

(G+H) Representative confocal images (G) and quantification (H) of HA levels in cell bodies of neurons stained with antibodies against HA and NeuN; 60/3 each.

(I and J) Representative areas of confocal images (I) and quantification (J) of HA levels in synapsin ROIs (the imaged areas are identical to the STED scans in Fig. 3F-H); 12/3 each.

Data are mean ± SEM; $^{***}p < 0.001$. Statistical significance compared to Ca_V2 cTKO was determined with Kruskal-Wallis tests followed by Dunn's multiple comparisons post-hoc tests for the protein of interest in C, E, H, and J.

bioRxiv preprint doi: https://doi.org/10.1101/2023.12.23.573183; this version posted December 23, 2023. The copyright holder for this preprint (which was not certified by peer review) is the author/funder, who has granted bioRxiv a license to display the preprint in perpetuity. It is made available under aCC-BY 4.0 International license.

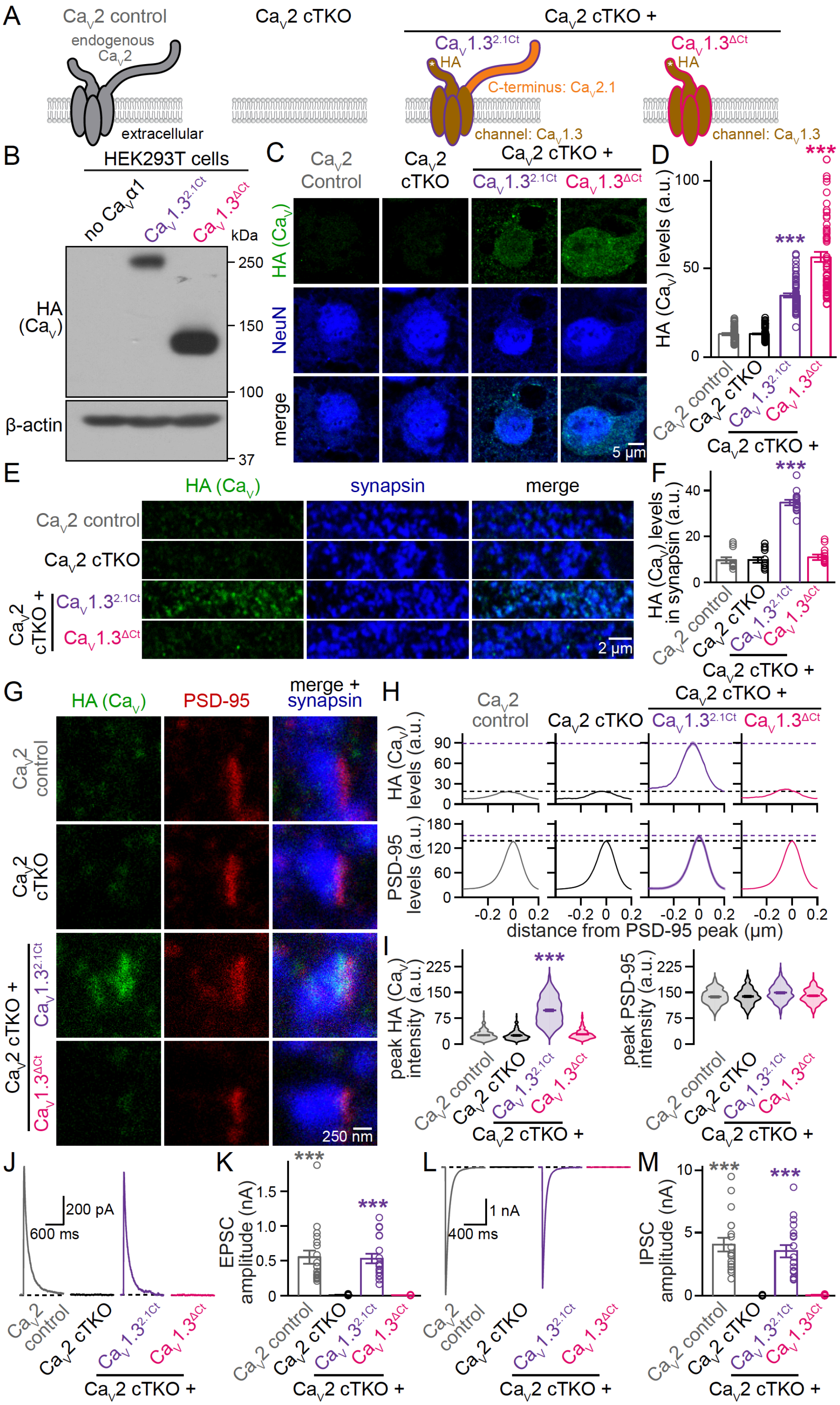

Figure S4. Assessment of Cav1.3$^{\Delta Ct}$.

bioRxiv preprint doi: https://doi.org/10.1101/2023.12.23.573183; this version posted December 23, 2023. The copyright holder for this preprint (which was not certified by peer review) is the author/funder, who has granted bioRxiv a license to display the preprint in perpetuity. It is made available under aCC-BY 4.0 International license.

(A) Schematic of the conditions for comparison.

(B) Western blot of HEK293T cell homogenates after transfection with $Ca_V\beta1$, $Ca_V\alpha2\delta1$, and without (no $Ca_V\alpha1$) or with a $Ca_V\alpha1$ subunit to assess $Ca_V\alpha1$ expression, a representative blot from two independent repeats is shown.

(C+D) Representative confocal images (C) and quantification (D) of HA levels in cell bodies of neurons stained with antibodies against HA and NeuN; 60 somata/3 independent cultures each.

(E+F) Representative areas of confocal images (E) and quantification (F) of HA levels in synapsin ROIs; Ca_V2 control, 11 images/3 independent cultures; Ca_V2 cTKO, 12/3; Ca_V2 cTKO + $Ca_V1.3^{2.1Ct}$, 14/3; Ca_V2 cTKO + $Ca_V1.3^{\Delta Ct}$, 14/3.

(G-I) Representative images (G) and summary plots of intensity profiles (H) and peak levels (I) of HA and PSD-95 at side-view synapses stained for HA (STED), PSD-95 (STED), and synapsin (confocal). The imaged areas are identical to the ones used for confocal analyses in E+F. Dashed lines in H denote levels in Ca_V2 cTKO (black) and Ca_V2 cTKO + $Ca_V1.3^{2.1Ct}$ (purple); Ca_V2 control, 198 synapses/3 independent cultures; Ca_V2 cTKO, 190/3; Ca_V2 cTKO + $Ca_V1.3^{2.1Ct}$, 207/3; Ca_V2 cTKO + $Ca_V1.3^{\Delta Ct}$, 195/3.

(J+K) Representative traces (J) and quantification (K) of NMDAR-mediated EPSCs; 18 cells/3 independent cultures each.

(L+M) As in J and K, but for IPSCs; 18/3 each.

Data are mean ± SEM; and ***$p < 0.001$. Statistical significance compared to Ca_V2 cTKO was determined with Kruskal-Wallis tests followed by Dunn's multiple comparisons post-hoc tests for the protein of interest or amplitudes in D, F, I, K, and M.

bioRxiv preprint doi: https://doi.org/10.1101/2023.12.23.573183; this version posted December 23, 2023. The copyright holder for this preprint (which was not certified by peer review) is the author/funder, who has granted bioRxiv a license to display the preprint in perpetuity. It is made available under aCC-BY 4.0 International license.

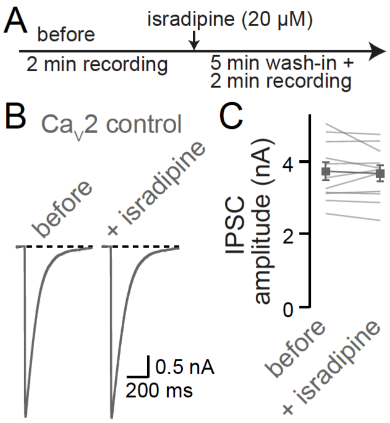

Figure S5. Assessment of L-type blocker sensitivity of synaptic transmission in Ca_V2 control neurons.

(A) Experimental strategy to evaluate blocker sensitivity of synaptic transmission.

(B+C) Representative traces (B) and quantification (C) of IPSCs recorded as outlined in A; 11 cells/3 independent cultures.

Data are mean ± SEM.
